# A systematic review of selected food-borne helminthiases in Southeast Asia with emphasis on anthelminthic treatment regimens

**DOI:** 10.1016/j.fawpar.2026.e00362

**Published:** 2026-09-13

**Authors:** Abigail Hui En Chan, Borimas Hanboonkunupakarn, Rachatawan Chiabchalard, Dorn Watthanakulpanich

**Affiliations:** a Department of Helminthology, Faculty of Tropical Medicine, Mahidol University, Thailand; b Department of Clinical Tropical Medicine, Faculty of Tropical Medicine, Mahidol University, Thailand; c Department of Protozoology, Faculty of Tropical Medicine, Mahidol University, Thailand

**Keywords:** Food-borne helminths, *Opisthorchis*, Southeast Asia, Systematic review, *Taenia*, Treatment

## Abstract

Food-borne helminth infections impose a substantial public health burden worldwide, particularly in tropical and subtropical regions. Environmental, socioeconomic, behavioral, and cultural factors all favor sustained transmission of food-borne helminths, especially in Southeast Asia (SEA). To provide an updated overview of the distribution, prevalence, and treatment regimens for six important food-borne helminths (*Opisthorchis viverrini*, *Fasciola* spp., *Taenia* spp., *Angiostrongylus* spp., *Gnathostoma spinigerum*, and *Trichinella* spp.) in SEA, a systematic review following the PRISMA guidelines was conducted. Anthelminthic drug treatment regimens based on the extracted studies were compared with those recommended by internationally recognized organizations. Of the 11 SEA countries, the highest proportion of peer-reviewed articles (47.2%) originated from Thailand, followed by Lao PDR (20.2%). All six helminths were reported in Vietnam, while five were found in Thailand. Meta-analyses were conducted for *O. viverrini*, taeniasis, and cysticercosis, as these had the greatest number of articles, and the overall pooled prevalences were 22.2%, 3.4%, and 4.7%, respectively. The country with the highest prevalence for *O. viverrini* infection and taeniasis was Lao PDR, with a prevalence of 43.2% and 3.6%, respectively. Drug treatment regimens reported in studies from SEA are in line with relevant international recommendations. However, the continued presence of food-borne helminthiases suggests sustained transmission despite appropriate treatment regimens. Moreover, the uneven distribution of studies across SEA reflects differences in research emphasis and diagnostic capabilities, highlighting the need for strengthened surveillance and coordinated efforts, especially in underrepresented countries. A One Health approach and regional collaborations will be critical for reducing the burden of food-borne helminthiases.

## 1 Introduction

Food-borne helminth infections continue to impose a substantial public health burden worldwide, particularly in tropical and subtropical regions where environmental, socioeconomic, behavioral, and cultural factors favor sustain transmission (Chávez-Ruvalcaba et al., 2021; Tidman et al., 2023). Food-borne helminths include species in Nematoda, Trematoda, and Cestoda, affecting more than one billion people globally (Maurice et al., 2020; Ortiz et al., 2024). According to the World Health Organization (WHO), the number of people infected with food-borne trematodes is estimated at 56 million, with 7000 deaths per year (World Health Organization, 2012). Cysticercosis, caused by the cestode *Taenia solium*, is estimated to infect 0.4 million people (World Health Organization, 2012). In addition, the Southeast Asia (SEA) region, comprising 11 countries (Thailand, Lao People's Democratic Republic, Myanmar, Vietnam, Cambodia, Indonesia, Malaysia, Philippines, Singapore, Brunei, and Timor Leste), is known to be a hotspot for food-borne helminth infections (Martviset et al., 2023a; Utzinger et al., 2015).

In SEA, traditional practices such as the consumption of raw or undercooked meat, fish, vegetables, and seafood increase the risk of infection. For example, traditional dishes incorporating raw or fermented fish are common in countries surrounding the Mekong basin, resulting in infections with the liver fluke *Opisthorchis viverrini* or minute intestinal flukes (Boondit et al., 2020; Khuntikeo et al., 2018; Sithithaworn et al., 2012; Sripa et al., 2010). Although primarily present in East Asia, the liver fluke *Clonorchis sinensis* is also very common in rural areas of North Vietnam, highlighting the medical importance of food-borne helminthiasis (Nguyen et al., 2020). Similarly, consuming raw or undercooked meat (beef or pork) is common in Vietnam, Indonesia, and Thailand, where raw meat may be seasoned with wine, lime juice, or spices (Eichenberger et al., 2020). Additionally, the communal eating behavior of communities in SEA may facilitate outbreaks (Kaewpitoon et al., 2008; Kusolsuk et al., 2010; Phimpraphai et al., 2018; Saenna et al., 2017).

Pharmacological treatment remains the cornerstone of helminthic control and patient management. Internationally recognized organizations such as the WHO (https://www.who.int/), Centers for Disease Control and Prevention (CDC) (https://www.cdc.gov/), and Médecins Sans Frontières (MSF) (https://www.msf.org/) provide guidance on recommended anthelmintic drugs and dosing regimens. However, in many endemic and resource-limited settings, frontline healthcare facilities face significant barriers to implementing these recommendations. These include irregular drug supply chains, limited national procurement mechanisms, and regulatory constraints. Consequently, availability of first-line anthelminthics may be limited, and clinicians are often compelled to use alternative drugs or modified dosing regimens that may not be optimal or explicitly endorsed by international guidelines (Sayasone et al., 2018; Velev et al., 2020). In addition, local treatment recommendations for helminth infections vary between and within countries, and treatment regimens may be insufficiently tailored to local epidemiological contexts. Moreover, the emergence of drug resistance, particularly to widely used anthelmintics such as triclabendazole and albendazole, further emphasizes the need to critically evaluate both standard and alternative treatment options.

Despite the regional impact of food-borne helminths on human health, food-borne helminthiases remain neglected and sustained transmission persists in SEA. Here, based on a systematic review of published data, we aim to provide an updated overview of the distribution, prevalence, and treatment regimens for six important food-borne helminths including *O. viverrini*, *Fasciola* spp., *Taenia* spp., *Angiostrongylus* spp., *Gnathostoma spinigerum*, and *Trichinella* spp. in SEA. *C. sinensis* was not included as within SEA, it is present only in Vietnam. Additionally, the drug treatments for these food-borne helminthiases recommended by WHO, CDC, and MSF were compared with the data reviewed and with those from the Hospital for Tropical Diseases, Faculty of Tropical Medicine, Mahidol University, Bangkok, Thailand. The information obtained will aid in making informed choices regarding treatment and control strategies, tailored to the regional context.

## 2 Materials and methods

### 2.1 Search strategy

Following the PRISMA guidelines (http://www.prisma-statement.org/) (Supplementary file 1), identification of publications on the six important food-borne helminths in SEA was carried out in the PubMed database in January 2026. These six helminths covering the three main helminth groups (trematodes, cestodes, and nematodes) were chosen based on their high prevalence in humans in SEA. The keywords used were: each of the helminth species – '*Opisthorchis viverrini'*, '*Fasciola'*, '*Taenia'*, '*Angiostrongylus'*, '*Gnathostoma spinigerum'*, '*Trichinella'* OR disease name – 'opisthorchiasis', 'fascioliasis', 'fasciolosis', 'taeniasis', 'taeniosis', 'cysticercosis', 'neurocysticercosis', 'angiostrongyliasis', 'gnathostomiasis', 'trichinellosis' AND each of the 11 countries in SEA – 'Thailand', 'Lao PDR', 'Vietnam', 'Cambodia', 'Myanmar', 'Indonesia', 'Malaysia', 'Singapore', 'Philippines', 'Brunei', 'Timor-Leste' AND 'Human'. The database search was conducted by two researchers, while the titles and abstracts were independently evaluated.

### 2.2 Exclusion and inclusion criteria

After identification in the PubMed database the articles were entered into a Microsoft Office Excel (Microsoft Cooperation, Albuquerque, USA) spreadsheet. Duplicate articles were removed and the articles were assessed on whether they should be included or excluded. This assessment was carried out by two independent reviewers and if there was a discrepancy in decision, the reviewers discussed whether to include or exclude the article. Articles were included if they 1) contained data on the presence of the specific helminth species in humans in any of the SEA countries, regardless of whether they contained information on drug treatment, 2) were published between 2015 and 2026, 3) were peer-reviewed research articles and case reports, and 4) included an abstract. Additionally, articles on migrant workers or travelers in SEA were also eligible for inclusion. Articles were excluded if they did not meet the inclusion criteria, i.e. if they were review articles, focused on the evaluation of diagnostic tests, described studies conducted on animals, and those articles that were not in the English language. Additionally, articles that only reported clinical features without diagnostic confirmation by either morphological, molecular, or serological techniques were not included.

### 2.3 Data extraction

Data were extracted from the full-text articles and grouped based on each helminth species. The data that were extracted from each included article were: first author name, year of publication, country and province/state of study, type of sample, diagnostic method used, total sample size, number of positive samples, prevalence, anthelminthic drug and dosage used, and recovery status. If the total number of positive samples or prevalence were not stated, either value was inferred from the total sample size. If more than one diagnostic method was used, the method with the highest reported prevalence was selected for meta-analysis.

### 2.4 Statistical approach and meta-analysis

Of the selected food-borne helminthiases, meta-analysis of estimated pooled prevalence was conducted for *O. viverrini* infection, taeniasis, and cysticercosis, for which sufficient prevalence data were available. For each, the overall pooled prevalence estimates (with 95% confidence intervals) were calculated using the alpha method for the random effects model based on the inverse variance method for measuring weight. Cochran Q test and the inconsistency index (I^2^) were used to assess the degree of heterogeneity among studies, with values more than 75% considered high heterogeneity. Publication bias was calculated using Egger's regression and Begg's test, where a *P*-value of <0.05 indicated the presence of publication bias.

The distribution of the six helminths in SEA was plotted on a map, and all statistics, meta-analysis, and map generation were done in R studio version 1.4.1717 (RStudio, PBC, Boston, USA).

### 2.5 Comparison of treatment regimes

Recommendations of anthelminthic drug usage and dosage for the treatment of each helminth infection by the WHO, CDC, and MSF were compiled and compared with the treatment regimens stated in the articles and local treatment practices at the Hospital for Tropical Diseases, Faculty of Tropical Medicine, Mahidol University, Bangkok, Thailand.

## 3 Results

### 3.1 Data characteristics and distribution of selected food-borne helminths in Southeast Asia

**Fig. 1:**
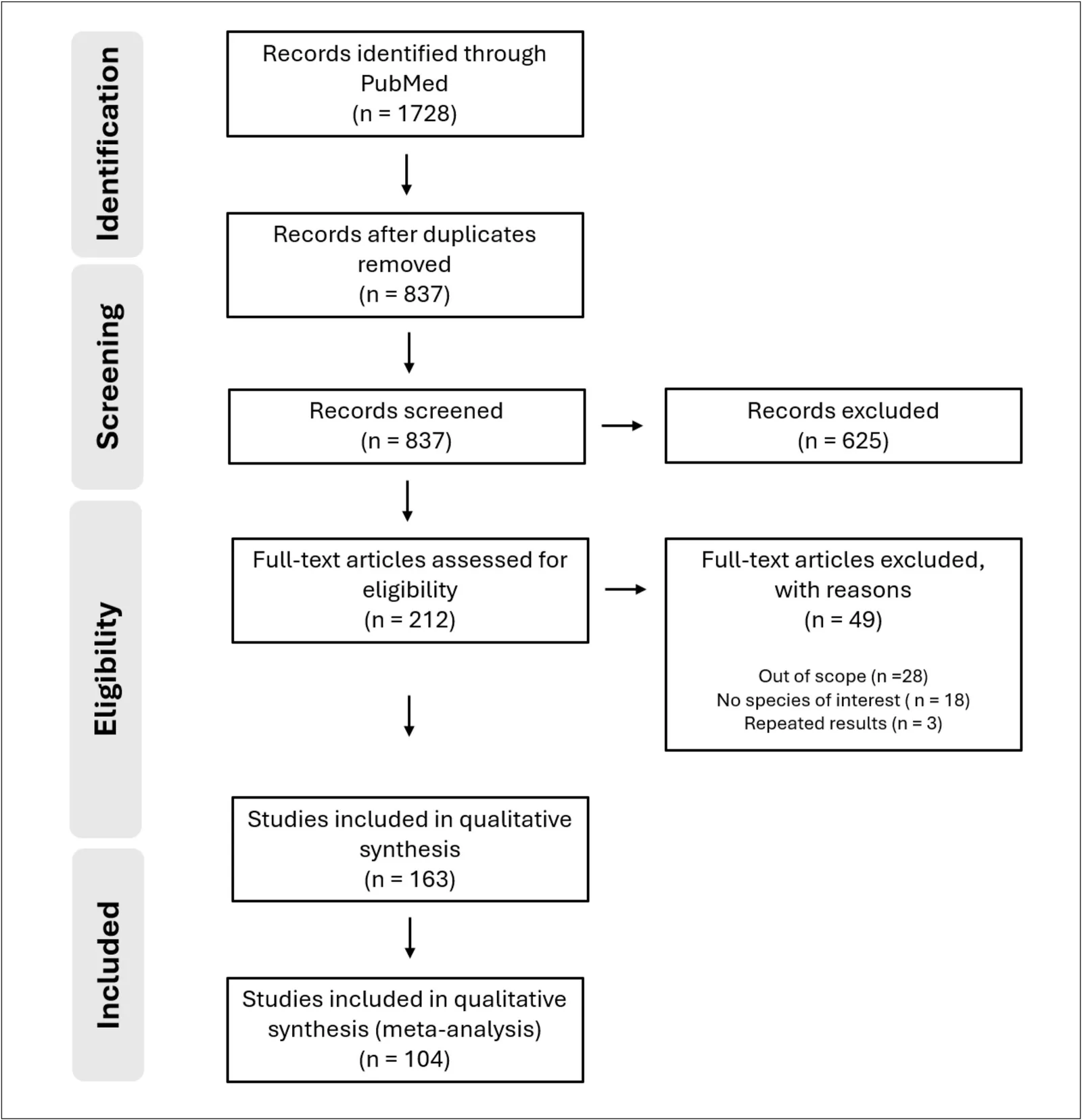
PRISMA flowchart of the search strategy used.

Using the PubMed database, a total of 1728 records were identified. Of these, 837 publications were obtained after the duplicates were removed. The titles and abstracts were then screened, resulting in 212 full-text articles assessed for eligibility. Of these, 49 articles were excluded, 28 for not being within the scope of this systematic review, 18 for not focusing on the relevant helminths, and three for presenting repeated results. The remaining 163 articles were included in qualitative synthesis, and 104 were used for meta-analysis. Meta-analysis was only conducted for *Opisthorchis* infection, taeniasis*,* and cysticercosis, as explained above. The PRISMA flow chart depicting the article selection process is presented in Fig. 1.

**Fig. 2:**
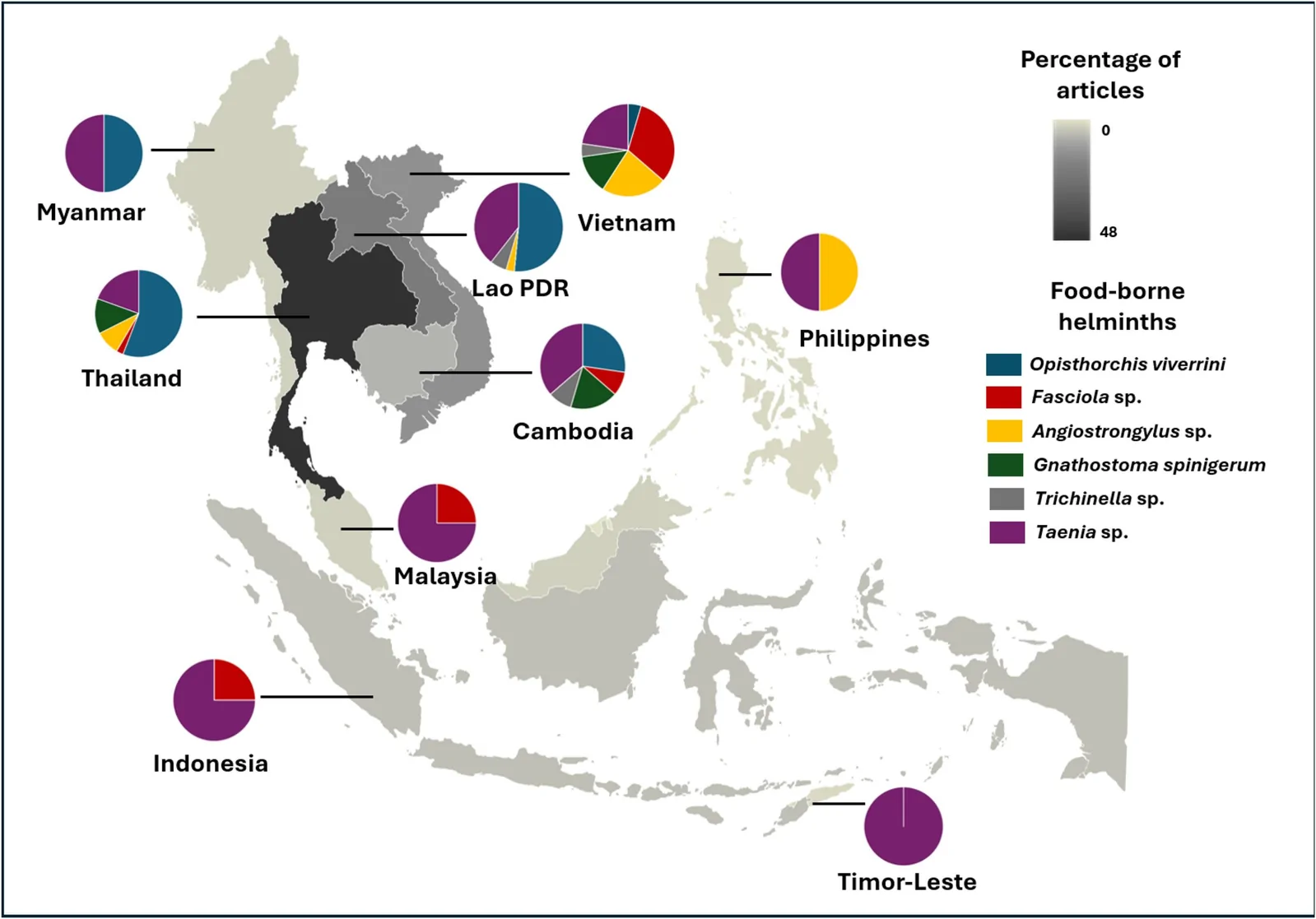
Distribution of selected food-borne helminths in Southeast Asia based on the articles in the last decade (2015 to 2026).

Of the 11 SEA countries, articles on the six food borne helminths in the last decade originated from nine countries – Cambodia, Indonesia, Lao PDR, Malaysia, Myanmar, Philippines, Thailand, Timor Leste, and Vietnam, while there were no articles from Brunei and Singapore. The highest proportion of articles came from Thailand (47.2%, 77 articles), followed by Lao PDR (20.2%, 33 articles), and Vietnam (13.5%, 22 articles). Fig. 2 presents the proportion of articles from each country, alongside the distribution of the food borne helminths included in this study. All six helminths were recorded in Vietnam, followed by five in Thailand and Cambodia. The five helminths recorded in Thailand included *O. viverrini*, *Fasciola* spp., *Angiostrongylus* spp., *G. spinigerum*, and *Taenia* spp., while the helminths recorded in Cambodia were *O. viverrini*, *Fasciola* spp., *G. spinigerum*, *Trichinella* spp., and *Taenia* spp. In Lao PDR, four helminths were recorded, while reports from Indonesia, Myanmar, Malaysia, and Philippines only reported two; finally, only one species was recorded in Timor Leste. Of the six helminth species, *Taenia* spp. was reported in all nine countries, while *O. viverrini* and *Fasciola* were found in five.

### 3.2 Prevalence of *O. viverrini* infection in Southeast Asia

**Fig. 3:**
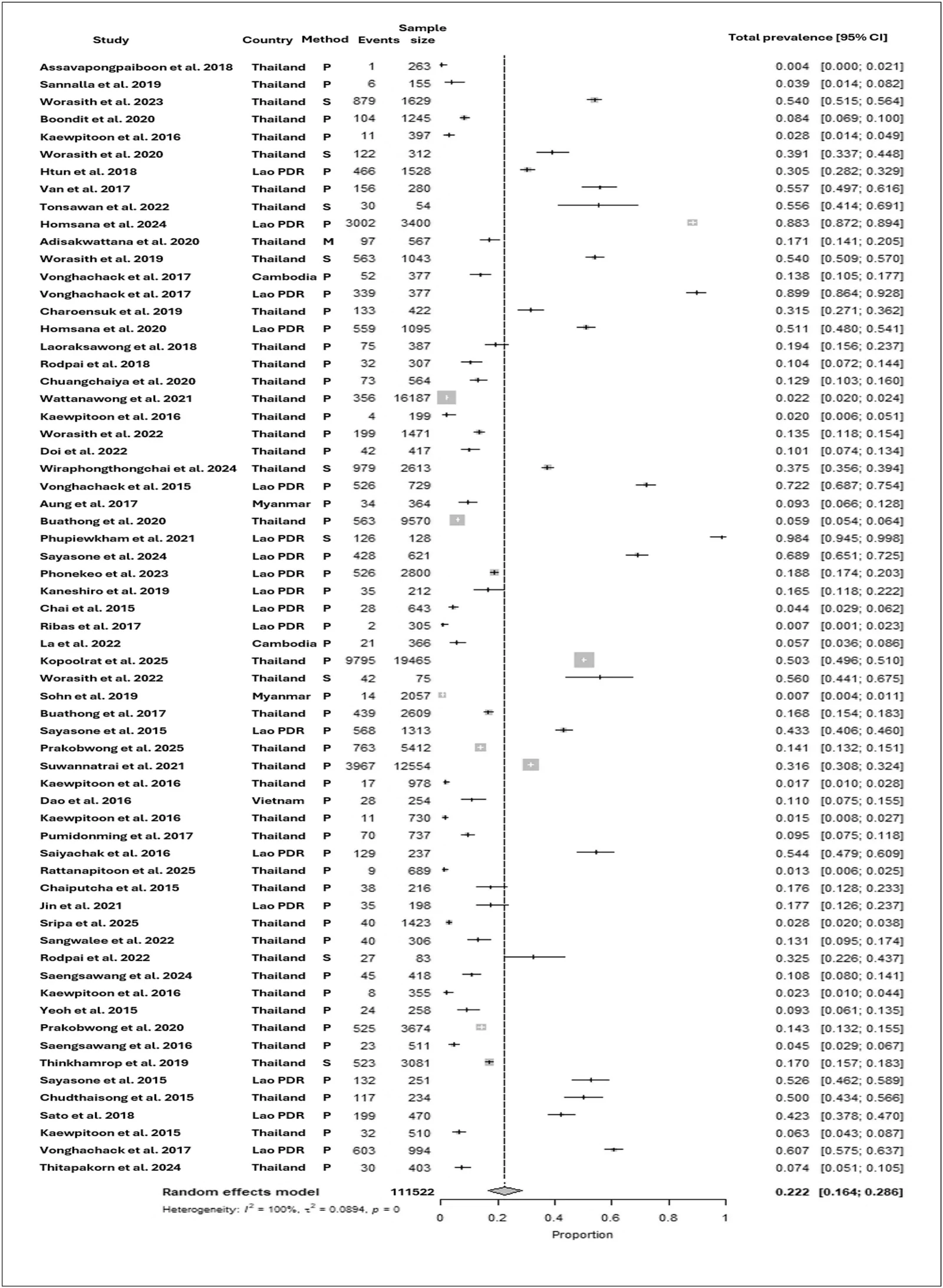
Forest plot of *Opisthorchis viverrini* infection prevalence in Southeast Asia based on the random effects model. The diagnostic techniques used in each study are abbreviated as P: parasitological, S: serological, and M: molecular.

A total of 64 studies on *O. viverrini* in SEA were included for meta-analysis (Supplementary file 2). The overall pooled prevalence of *O. viverrini* infection based on the random effects model was 22.2% (95% CI 16.4–28.6%), with majority of the studies conducted in Thailand (Fig. 3). The Cochran Q test (*p* < 0.0001) and I^2^ index revealed a high level of heterogeneity (99.8%) among the studies. Low publication bias was indicated by Egger's test (*p* = 0.902) and Begg's test (*p* = 0.225). The Kato-Katz and formalin-ether concentration techniques were the most commonly used diagnostic methods, comprising 54 (84.4%) studies. Nine (14.1%) studies were based on serological diagnosis, while one (1.5%) used molecular techniques.

Subgroup analysis for Thailand based on 40 studies revealed an estimated pooled prevalence of 17.2% (95% CI 11.3–24.1%), which was slightly lower than the overall pooled prevalence in SEA. Cochran Q test (*p* < 0.0001) and I^2^ index (99.8%) indicated a high level of heterogeneity, while both Egger's test (*p* = 0.520) and Begg's test (*p* = 0.351) showed low publication bias. In contrast, the estimated pooled prevalence obtained for Lao PDR was 43.2% (95% CI 26.4–60.9%), higher than the overall pooled prevalence in SEA. High level of heterogeneity (*p* < 0.0001 Cochran Q test, 99.8% I^2^ index) and low publication bias (*p* = 0.457 Egger's test and *p* = 0.621 Begg's test) were obtained. Subgroup analysis was not performed for Cambodia, Myanmar, and Vietnam as there were only five studies shared among them. The prevalence of *O. viverrini* infection ranged from 5.7 to 13.8% in Cambodia and from 0.7 to 9.3% in Myanmar, while it was 11.4% in Vietnam.

### 3.3 Prevalence of taeniasis in Southeast Asia

**Fig. 4:**
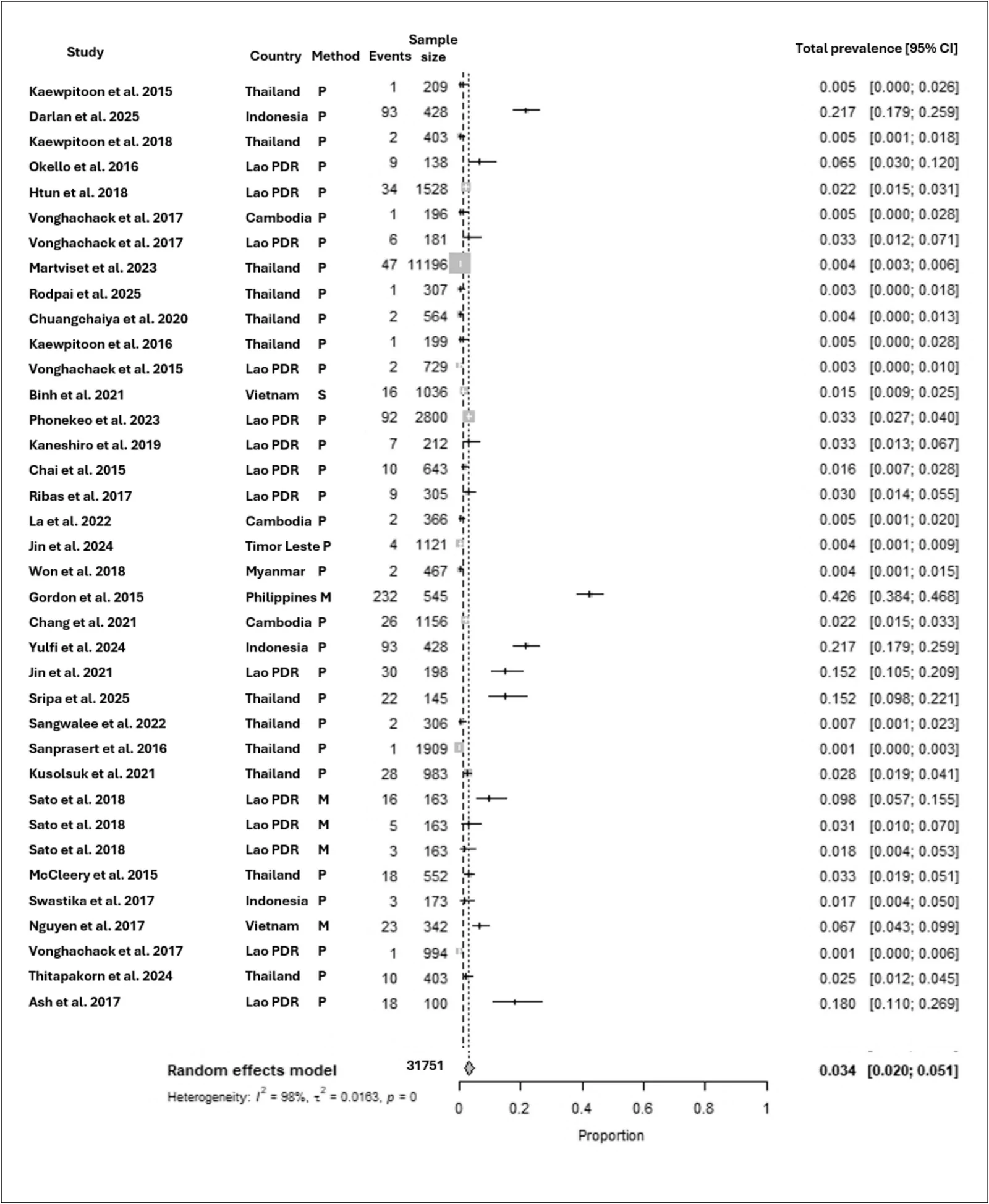
Forest plot of taeniasis prevalence in Southeast Asia based on the random effects model. The diagnostic techniques used in each study are abbreviated as P: parasitological, S: serological, and M: molecular.

Of the 35 studies on taeniasis, the overall pooled prevalence in SEA using the random effects model was 3.4% (95% CI 1.9–5.0%) (Fig. 4 and Supplementary file 3). The Cochran Q test (*p* < 0.0001) and I^2^ (98.0%) revealed a high degree of heterogeneity, while both Egger's test (*p* = 0.006) and Begg's test (*p* = 0.017) showed substantial publication bias. *Taenia* spp. was studied in nine countries, with the highest number of studies conducted in Lao PDR (*n* = 14), followed by Thailand (*n* = 12). Of the 35 studies, 31 employed parasitological techniques to detect *Taenia* spp. eggs in fecal samples, three used copro-PCR for direct molecular identification, while one study used copro-Ag to detect *Taenia* antigen in fecal samples.

Subgroup analysis revealed an estimated pooled prevalence of 3.6% for *Taenia* infection in Lao PDR (95% CI 2.0–5.7%). Heterogeneity was high, as indicated by the Cochran Q test (*p* < 0.0001) and I^2^ (93.6%). In contrast, publication bias was low, as indicated by both Egger's test (*p* = 0.155) and Begg's test (*p* = 0.058). In Thailand, the estimated pooled prevalence was 1.2% (95% CI 0.5–2.3%), lower than the overall pooled prevalence in SEA. Heterogeneity was high here too (Cochran Q test *p* < 0.001; I^2^ 93.1%, whereas publication bias was low according to both Egger's test (*p* = 0.072) and Begg's test (*p* = 0.146). Subgroup analyses were not performed for the remaining seven countries because of the limited number of available studies. However, individual studies from the Philippines and Indonesia reported high prevalences of *Taenia* infection, at 42.5% and 21.7%, respectively.

Three species of *Taenia* were present in SEA, comprising of *T. solium*, *T. saginata*, and *T. asiatica*. *Taenia solium* was present in Thailand, Lao PDR, Timor Leste, Indonesia, and Vietnam, while *T. saginata* was found in Lao PDR, Myanmar, Philippines, Thailand, Indonesia, and Vietnam. *Taenia asiatica* was identified in Indonesia, Lao PDR, Vietnam, and Thailand.

### 3.4 Prevalence of cysticercosis in SEA

**Fig. 5:**
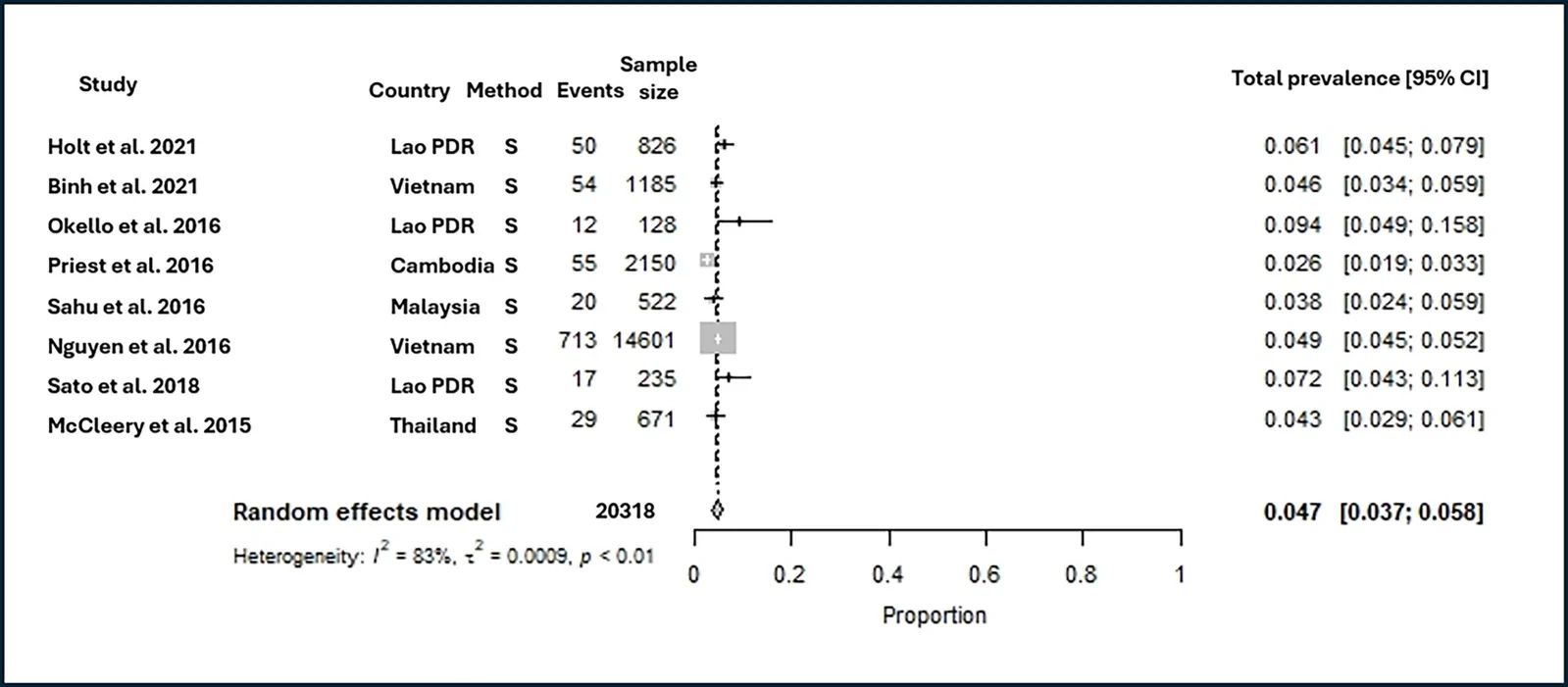
Forest plot of cysticercosis prevalence in Southeast Asia based on the random effects model. The diagnostic techniques used in each study are abbreviated as P: parasitological, S: serological, and M: molecular.

For cysticercosis, prevalence data were available from eight studies. The overall pooled prevalence using the random effects model was 4.7% (95% CI 3.6–5.8%) (Fig. 5 and Supplementary file 3). Cochran Q test (*p* < 0.0001) and I^2^ (83.1%) revealed a high degree of heterogeneity, while Egger's test (*p* = 0.865) and Begg's test (*p* = 0.804) showed low publication bias. Cysticercosis was studied in five countries, with three studies from Lao PDR, two from Vietnam, and one each from Cambodia, Thailand, and Malaysia. In all eight studies, the diagnosis of cysticercosis was based on serological testing.

For neurocysticercosis, a total of six studies was identified, originating from Malaysia, Indonesia, Timor Leste, Thailand, Vietnam, and Myanmar (Supplementary file 3). Diagnostic methods included serological testing of serum or cerebrospinal fluid, cyst identification, and molecular identification of excised cysts.

### 3.5 Other food-borne helminthiases

*Fasciola* infection was reported in 12 publications from Thailand, Indonesia, Vietnam, Cambodia, and Malaysia (Supplementary File 4). Seven of the reports were from Vietnam, while two were from Thailand. Additional cases were reported in travelers from Germany, France, and Australia, following travel to Thailand, Vietnam, and Indonesia, respectively. The species of *Fasciola* reported in SEA included *Fasciola hepatica, Fasciola gigantica*, and their hybrid forms. Serological methods based on IgG detection by ELISA or western blot were used in nine of the studies. Triclabendazole was the only drug reported for the treatment of fascioliasis.

For *Angiostrongylus*, a total of 14 publications were identified, originating from Thailand, Vietnam, Lao PDR, and the Philippines. *Angiostrongylus cantonensis* was the predominant causative agent, with one report of a mixed *A. cantonensis* and *Angiostrongylus malaysiensis* infection in Thailand. Both serological and molecular methods were used for diagnosis, while treatment consisted of albendazole and/or corticosteroids. Three cases of ocular angiostrongyliasis involving surgical removal of the worm were reported.

*Gnathostoma spinigerum* was reported in 15 publications from Thailand, Vietnam, and Cambodia. Of these, 10 were from Thailand, three were from Vietnam and two from Cambodia. The diagnostic methods included morphological identification following worm removal and serology. Treatment involved albendazole, ivermectin, or a combination of both drugs.

The fewest publications were identified for *Trichinella,* with reports from Lao PDR, Cambodia, and Vietnam. *Trichinella spiralis* and *Trichinella papuae* were the causative species. All studies used serological methods for diagnosis, and albendazole was the drug of choice.

### 3.6 Anthelminthic drug treatment regimens

Comparison of treatment regimens for the analyzed food-borne helminthiases showed that those reported in the reviewed publications were generally consistent with recommendations from WHO, CDC, and MSF (Table 1). For *O. viverrini* infection, praziquantel and albendazole are both recommended by CDC and the Hospital for Tropical Diseases, Mahidol University. For *Fasciola* infection, triclabendazole is recommended by WHO, CDC, and MSF, and was the only drug reported in the reviewed publications. For angiostrongyliasis, supportive treatment is recommended by the CDC, whereas albendazole and surgical removal were reported in the reviewed publications. For gnathostomiasis, both albendazole and ivermectin are recommended by the CDC and the Hospital for Tropical Diseases, Mahidol University, and the reviewed publications reported the use of either drug alone or in combination. For *Trichinella* infection, both CDC and the Hospital for Tropical Diseases, Mahidol University recommend albendazole and mebendazole. For taeniasis, praziquantel and niclosamide are recommended by the CDC, WHO, and MSF, while the use of albendazole was also reported in the reviewed publications. For cysticercosis, treatment recommendations were available from both WHO and CDC, and the treatment regimens reported in the reviewed publications were generally consistent with these recommendations.

**Table 1:** Comparison of anthelminthic drug treatment regimes.

Helminth	WHO	CDC	MSF	Articles	Hospital for Tropical Diseases, Mahidol University
*O. viverrini*	1. Praziquantel: - 75 mg/kg/day divided into 3 doses for 1 day - 40 mg/kg single dose	1. Praziquantel: - 75 mg/kg/day divided into 3 doses for 2 days 2. Albendazole: - 10 mg/kg/day for 7 days	NA	1. Praziquantel: - 40 mg/kg single dose	1. Praziquantel: - 25 mg/kg/day divided into 3 doses for 2 days - 40 mg/kg single dose 2. Albendazole: - 10 mg/kg/day for 7 days
*Fasciola* spp.	1. Triclabendazole: - 10 mg/kg single dose	1. Triclabendazole: - 10 mg/kg two doses, 12 h apart	1. Triclabendazole: - 10 mg/kg single dose	1. Triclabendazole: - 10 to 20 mg/kg two doses	Triclabendazole is not available in the country
*Angiostrongylus* spp.	NA	Supportive treatment for pain and inflammation	NA	1. Albendazole: - 800 mg/day for 5 days or 4 weeks - 200 mg twice a day for 10 days - 200 mg/day for 2 weeks - 600 mg twice a day - 15 mg/kg for 15 days 2. Surgical removal	1. Albendazole: - 200 mg twice a day for 10 days
*G. spinigerum*	NA	1. Albendazole: - 400 mg once or twice a day for 21 days 2. Ivermectin: - 200 μg/kg single dose or for 2 days	NA	1. Albendazole: - 15 mg/kg for 21 days - 400 mg twice a day for 5 days 2. Albendazole + removal: - 400 mg/day for 3 weeks 3. Albendazole + Ivermectin: - albendazole 400 mg twice a day for 3 weeks + ivermectin 200 μg/kg/day for 2 days 4. Ivermectin: - 200 μg/kg 5.Surgical removal	1. Albendazole: - 400 mg twice a day for 21 days 2. Ivermectin: −200 μg/kg/day 2 days consecutively
*Trichinella* spp.	1. Albendazole: - 400 mg twice a day for 10 to 15 days. Treatment may be repeated after 5 days (in severe cases). 2. Mebendazole: 5 mg/kg per day divided in two doses for 10 to 15 days. The whole treatment cycle may be repeated after 5 days.	1. Albendazole: - 400 mg twice a day for 8 to 14 days 2. Mebendazole: - 200 to 400 mg 3 times a day for 3 days, then 400 to 500 mg three times a day for 10 days	1. Albendazole: - 400 mg twice a day for 10 to 15 days 2. Mebendazole: - 200 mg twice a day for 10 to 15 days	1. Albendazole: - 800 mg for 10 days −15 mg/kg for 10 days - 400 mg for 14 days	1. Albendazole: - 400 mg twice a day for 14 days 2. Mebendazole: - 200-400 mg three times a day for 3 days, then 500 mg three times a day for 10 days
*T. solium*/*T. saginata* (intestinal)	1. Praziquantel: - 5 to 10 mg/kg 2. Niclosamide: - 2 g	1. Praziquantel: - 5 to 10 mg/kg 2. Niclosamide: - 2 g	1. Praziquantel: - 5 to 10 mg/kg 2. Niclosamide: - 2 g	1. Praziquantel: - 10 to 40 mg/kg 2. Niclosamide: - 500 mg 3. Albendazole: - 400 mg	1. Praziquantel: - 20 mg/kg 2. Niclosamide: - 2 g single dose
*T. solium* (cysticercosis)	1. Surgery and/or long courses with praziquantel and/or albendazole, with supporting therapy with corticosteroids	1. Combination of anti-parasitic drugs and anti-inflammatory drugs, or surgery	NA	1. Praziquantel: - 50 mg/kg/day for 15 days 2. Praziquantel + Albendazole: - 400 to 600 mg + 400 mg twice/day	1. Albendazole: - 15 mg/kg/day in 2 divided doses for 10–14 days 2. Praziquantel: - 50 mg/kg/day for 10–14 days

## 4 Discussion

This systematic review and meta-analysis revealed substantial geographical variation in both the number of studies and the prevalence of helminth infections across SEA. Of all the studies identified, Thailand contributed the largest share, accounting for nearly half of them. The greatest number of studies were on *O. viverrini* and *Taenia* spp., demonstrating their importance and endemicity in the region.

### 4.1 Research disparities on food-borne helminthiases in Southeast Asia

The uneven distribution of studies across SEA reflects differences in research emphasis, surveillance, laboratory and diagnostic capabilities, highlighting the need for coordinated efforts especially in underrepresented countries. Thailand accounted for the highest proportion of included studies, which may reflect its strong research focus and surveillance efforts regarding food-borne helminthiases (Adisakwattana et al., 2020; Assavapongpaiboon et al., 2018; Chuangchaiya et al., 2020). Additionally, developing robust surveillance systems and diagnostic capabilities requires time. As a regional forerunner in these areas, Thailand has likely benefited from higher rates of infection detection and reporting (Martviset et al., 2023b; Wattanawong et al., 2021). In contrast, the low number of studies in Myanmar, Cambodia, Indonesia, the Philippines, and Timor-Leste likely reflects insufficient surveillance rather than low endemicity. Difficulties in accessing communities and healthcare services may hinder surveillance and data collection, thereby masking the true disease burden (Quinn et al., 2014; Swe et al., 2023). On the other hand, the absence of studies from Brunei and Singapore likely reflects an absence of helminth infections, which may be due to dietary habits, improved infrastructure and rapid economic development, and high hygiene standards (Chan et al., 2023).

### 4.2 *Opisthorchis viverrini* remains a public health concern

The persistence of *O. viverrini* in the Greater Mekong Region likely reflects shared food habits that facilitate sustained transmission (Forrer et al., 2012; Sripa et al., 2021; Tan and Machrumnizar, 2023). As compared to Thailand, a markedly high prevalence was observed in Lao PDR. The difference may be attributed to a lack of education and awareness regarding the health risks. In Thailand, surveillance and control of *O. viverrini* has been conducted for many decades (since the 1990s), which resulted in a significant decrease in infection prevalence (Buathong et al., 2024; Sota et al., 2024). Additionally, control strategies including environmental management, mass drug administration, health education campaigns and community engagement, increasing awareness as well as promoting behavior changes in food consumption have been put in place, especially in endemic provinces in northeastern Thailand (Charoensuk et al., 2024; Sripa et al., 2015). Recently, integrated One Health approaches have been implemented in endemic areas, targeting intermediate and reservoir hosts, as well as environmental determinants to further reduce infection prevalence and reinfection rates (Prakobwong et al., 2025). However, despite these measures and significant improvements, *O. viverrini* remains a public health concern in Thailand. Additionally, the limited use of serological and molecular techniques for the diagnosis of opisthorchiasis in SEA remains an important knowledge gap and may contribute to underestimation of its true prevalence.

### 4.3 Epidemiological complexity of *Taenia* spp.

Compared to *O. viverrini*, the pooled prevalence of *Taenia* spp. infection in SEA was comparatively lower. However, the distribution of *Taenia* spp. is broader than *O. viverrini*, present in nine countries, and is not restricted to the countries in the Greater Mekong Region. The highest infection prevalence was again obtained in Lao PDR, reflecting the country's burden of food-borne helminthiases. Moreover, transmission hotspots in Lao PDR are likely linked to the cultural practices of certain ethnic groups, where raw pork consumption is favored (Anantaphruti, 2013; Jeon et al., 2013). *Taenia* spp. has also been reported from border areas and hill tribes, where transmission is sustained through food consumption behavior, inadequate sanitation practices, and pig husbandry as the main income source (Ash et al., 2017; Okello et al., 2014). *Taenia solium*, *T. saginata*, and *T. asiatica* are all present in SEA, highlighting transmission via different sources (e.g. consumption of pork and visceral organs, and beef) (Binh et al., 2021; Holt et al., 2016; Kusolsuk et al., 2021; Sahu et al., 2016). Moreover, hybrids between *Taenia* species have been reported despite the parent species not sharing the same intermediate hosts (pig and cattle) (Chang et al., 2021; Sato et al., 2018). The occurrence of hybrids emphasizes the role of humans as an environment for genetic material exchange between *Taenia* species.

The epidemiological complexity and clinical significance of *Taenia* spp. is further compounded by cysticercosis and neurocysticercosis. Although cysticercosis is prevalent, often linked to places where pig farming is practiced, there is a scarcity of data in SEA (Bustos et al., 2026). Only eight and six studies on cysticercosis and neurocysticercosis, respectively, were identified (Supplementary File 3). For neurocysticercosis, Susilawathi et al., 2020 reported a total of 29 cases from Indonesia and Timor Leste from 2014 to 2018, with the number of cases declining over the years (Susilawathi et al., 2020). Although severe and potentially life threatening, a definite diagnosis is challenging based on clinical symptoms alone (Bustos et al., 2026). The diagnosis relies on neuroimaging, supported with serological and molecular tests (Almeida et al., 2006; Garcia et al., 2000; Toribio et al., 2024). These techniques may not be universally available in regions with limited infrastructure and resources, thus limiting diagnostic accuracy and coverage.

### 4.4 Increasing need to incorporate molecular diagnostics

Traditional parasitological microscopy-based methods remain the mainstay for diagnosis of food-borne helminthiases in SEA. Prevalence estimates for *O. viverrini* and *Taenia* spp. infections are largely influenced by the diagnostic methods employed and would likely be higher if molecular or serological methods were used more frequently than parasitological techniques. The latter are widely used in resource-limited environments due to their feasibility and low cost as they do not require complex laboratory equipment. However, the sensitivity of parasitological methods to detect low-intensity infections remains a limitation, masking the true prevalence (Shchelkanov et al., 2025). Serological methods, on the other hand, are widely used for tissue helminths (e.g. *Gnathostoma*, *Trichinella*, *Angiostrongylus*). However, the actual prevalence may be overestimated through the detection of past exposure rather than current infection (Parija and Poddar, 2025). Molecular diagnostics is preferred due to its high sensitivity, ability to detect current infections, and species-specific identification (Afresham et al., 2025; O'Connell and Nutman, 2016). Molecular methods also allow for the identification of hybrids, such as those identified in SEA among *Fasciola* (*F. hepatica* and *F. gigantica*), *Angiostrongylus* (*A. cantonensis* and *A. malaysiensis*), and *Taenia* spp. (Kaenkaew et al., 2024; Le et al., 2008; Sato et al., 2018).

### 4.5 Drug treatment in the context of ongoing endemicity in Southeast Asia

Anthelminthic drug treatment was generally consistent with recommendations from relevant international institutions, indicating that the guidelines have been broadly applied in SEA. However, variations in dosing regimens and combination therapies were observed, especially for gnathostomiasis and angiostrongyliasis. Also, the recommended treatment for neurocysticercosis remains non-specific, where regimens are highly dependent on the physician's expertise and patient's response. The variability in regimens highlights a need for more detailed guidelines, which will be especially valuable in endemic and hard-to-reach areas where experienced clinicians are not readily available. More importantly, inconsistent dosing regimens may create conditions for treatment failure and inadequate parasite clearance, allowing possible transmission (Geerts and Gryseels, 2000).

Despite appropriate drugs being administered, the continued presence of food-borne helminthiases suggests sustained transmission in endemic countries. Animal and environmental reservoirs, cultural practices involving consuming raw or undercooked food, and low disease awareness contribute to ongoing transmission and disease burden (Gabriël et al., 2022; Murrell, 2013). While effective and appropriate treatment remains essential for clinical management, it is crucial to interrupt transmission across the human-animal-environment interface. The treatment of reservoir hosts in drug administration programs is recommended, especially in areas where human to animal contact is frequent. Integrating these strategies, alongside those that reinforce behavioral changes, food safety practices, and health education are essential for sustainable reduction in transmission.

Although anthelmintic resistance has not been widely documented in food-borne helminthiases, it represents a potential concern in endemic settings. Reports of resistance in other human and animal helminths, including triclabendazole-resistant *F. hepatica* in humans, highlight the importance of continued surveillance of treatment efficacy (Babják et al., 2023; Cabada et al., 2016; Fairweather et al., 2020; Kelley et al., 2016; Kotze and Prichard, 2016; Morales et al., 2021).

### 4.6 Limitations

Limitations of this study include the limited number of studies available *Angiostrongylus*, *G. spinigerum*, *Trichinella*, and *Fasciola*, precluding meta-analysis. Prevalence estimates could not be generated for all countries, and further subgroup analyses by diagnostic method were not possible because of the small number of studies. In addition, the literature search was limited to a single database (PubMed).

## 5 Conclusions

The continued presence of food-borne helminthiases in SEA remains a public health concern. This review highlights persistent transmission across the region, alongside important gaps in epidemiological data and inconsistencies arising from differences in diagnostic methodologies. Strengthening surveillance systems and improving access to sensitive and standardized diagnostic approaches will be important for obtaining more accurate estimates of disease burden and informing control efforts.

The following are the supplementary data related to this article.

Supplementary material 1 PRISMA 2020 checklist.

Supplementary material 2 *Opisthorchis viverrini* data extracted from included studies.

Supplementary material 3 *Taenia* data extracted from included studies.

Supplementary material 4 Data from other food-borne helminths not included in meta-analysis.

## Authors' contribution

AC and DW conceptualized the manuscript. AC, BH, and RC collected, analyzed, and interpreted the data. AC wrote the original draft. All authors read and approved the final manuscript.

## CRediT authorship contribution statement

**Abigail Hui En Chan:** Writing – original draft, Investigation, Formal analysis, Conceptualization. **Borimas Hanboonkunupakarn:** Writing – review & editing, Investigation, Formal analysis. **Rachatawan Chiabchalard:** Writing – review & editing, Investigation, Formal analysis. **Dorn Watthanakulpanich:** Writing – review & editing, Conceptualization.

## Consent for publication

Not applicable.

## Ethics approval and consent to participate

Not applicable.

## Funding

No funding was obtained.

## Declaration of competing interest

The authors declare that they have no known competing financial interests or personal relationships that could have appeared to influence the work reported in this paper.

## Data Availability

All data generated or analyzed during this study are included in this published article and its supplementary information files. The review and protocol of this systematic review was not registered.

## References

[bb0005] Adisakwattana P., Yoonuan T., Phuphisut O., Poodeepiyasawat A., Homsuwan N., Gordon C.A. (2020). Clinical helminthiases in Thailand border regions show elevated prevalence levels using qPCR diagnostics combined with traditional microscopic methods. Parasit. Vectors.

[bb0010] Afresham S., Khan M.K., Mughal M.A.S., Mehmood M.S., Ali S., Bashir M. (2025). Recent advancements in the diagnosis of parasitic diseases. Mol. Biochem. Parasitol..

[bb0015] Almeida C.R., Ojopi E.P., Nunes C.M., Machado L.R., Takayanagui O.M., Livramento J.A. (2006). *Taenia solium* DNA is present in the cerebrospinal fluid of neurocysticercosis patients and can be used for diagnosis. Eur. Arch. Psychiatry Clin. Neurosci..

[bb0020] Anantaphruti M.T. (2013). Current status of taeniasis in Thailand. Korean J. Parasitol..

[bb0030] Ash A., Okello A., Khamlome B., Inthavong P., Allen J., Thompson R.C.A. (2017). Controlling *Taenia solium* and soil transmitted helminths in a northern Lao PDR village: impact of a triple dose albendazole regime. Acta Trop..

[bb0035] Assavapongpaiboon B., Bunkasem U., Sanprasert V., Nuchprayoon S. (2018). A cross-sectional study on intestinal parasitic infections in children in suburban public primary schools, Saraburi, the central region of Thailand. Am. J. Trop. Med. Hyg..

[bb0040] Babják M., Königová A., Komáromyová M., Kuzmina T., Nosal P., Várady M. (2023). Multidrug resistance in *Haemonchus contortus* in sheep - can it be overcome?. J. Vet. Res..

[bb0045] Binh V.T.L., Dung D.T., Vinh H.Q., Anke V.H., Nicolas P., Pierre D. (2021). Human taeniasis and cysticercosis and related factors in Phu Tho Province, Northern Vietnam. Korean J. Parasitol..

[bb0050] Boondit J., Suwannahitatorn P., Siripattanapipong S., Leelayoova S., Mungthin M., Tan-ariya P. (2020). An epidemiological survey of *Opisthorchis viverrini* infection in a lightly infected community, eastern Thailand. Am. J. Trop. Med. Hyg..

[bb0055] Buathong S., Lakhanawan C., Suwannahitatorn P. (2024). The past and present situation of *Opisthorchis viverrini* infection in Thailand. Vajira Med. J..

[bb0060] Bustos J.A., Coyle C.M., Thakur K.T., Guzman C., Toribio L.M., Arroyo G. (2026). *Taenia solium* neurocysticercosis: its current epidemiological, diagnostic, therapeutic, and control landscapes. PLoS Negl. Trop. Dis..

[bb0065] Cabada M.M., Lopez M., Cruz M., Delgado J.R., Hill V., White A.C. (2016). Treatment failure after multiple courses of triclabendazole among patients with fascioliasis in Cusco, Peru: a case series. PLoS Negl. Trop. Dis..

[bb0070] Chan A.H.E., Kusolsuk T., Watthanakulpanich D., Pakdee W., Doanh P.N., Yasin A.M. (2023). Prevalence of *Strongyloides* in Southeast Asia: a systematic review and meta-analysis with implications for public health and sustainable control strategies. Infect. Dis. Poverty.

[bb0075] Chang T., Jung B.K., Hong S., Shin H., Ryoo S., Lee J. (2021). Occurrence of a hybrid between *Taenia saginata* and *Taenia asiatica* tapeworms in Cambodia. Korean J. Parasitol..

[bb0080] Charoensuk L., Chedtabud K., Chaipibool S., Laothong U., Suwannatrai A., Pinlaor S. (2024). Integrated one-health approach for prevention and control of *Opisthorchis viverrini* infection in rural Thailand: a 3-year study. Parasitol. Res..

[bb0085] Chávez-Ruvalcaba F., Chávez-Ruvalcaba M., Moran Santibañez K., Muñoz-Carrillo J., León Coria A., Reyna Martínez R. (2021). Foodborne parasitic diseases in the neotropics – a review. Helminthologia.

[bb0090] Chuangchaiya S., Navanesan S., Jaichuang S., Rahim M.A.F.A., Idris Z.M. (2020). Current prevalence of *Opisthorchis viverrini* infection and associated risk factors in Nakhon Phanom Province, Northeastern Thailand. Trop. Biomed..

[bb0100] Eichenberger R.M., Thomas L.F., Gabriël S., Bobić B., Devleesschauwer B., Robertson L.J. (2020). Epidemiology of *Taenia saginata* taeniosis/cysticercosis: a systematic review of the distribution in east, Southeast and South Asia. Parasit. Vectors.

[bb0105] Fairweather I., Brennan G.P., Hanna R.E.B., Robinson M.W., Skuce P.J. (2020). Drug resistance in liver flukes. Int. J. Parasitol. Drugs Drug Resist..

[bb0110] Forrer A., Sayasone S., Vounatsou P., Vonghachack Y., Bouakhasith D., Vogt S. (2012). Spatial distribution of, and risk factors for, *Opisthorchis viverrini* infection in southern Lao PDR. PLoS Negl. Trop. Dis..

[bb0115] Gabriël S., Dorny P., Saelens G., Dermauw V. (2022). Foodborne parasites and their complex life cycles challenging food safety in different food chains. Foods.

[bb0120] Garcia H.H., Parkhouse R.M., Cilman R.H., Montenegro T., Bernal T., Martinez S.M. (2000). Serum antigen detection in the diagnosis, treatment, and follow-up of neurocysticercosis patients. Trans. R. Soc. Trop. Med. Hyg..

[bb0125] Geerts S., Gryseels B. (2000). Drug resistance in human helminths: current situation and lessons from livestock. Clin. Microbiol. Rev..

[bb0130] Holt H.R., Inthavong P., Khamlome B., Blaszak K., Keokamphe C., Somoulay V. (2016). Endemicity of zoonotic diseases in pigs and humans in lowland and upland Lao PDR: identification of socio-cultural risk factors. PLoS Negl. Trop. Dis..

[bb0135] Jeon H.K., Yong T.S., Sohn W.M., Chai J.Y., Min D.Y., Yun C.H. (2013). Current status of human taeniasis in Lao People’s Democratic Republic. Korean J. Parasitol..

[bb0140] Kaenkaew C., Chan A.H.E., Saralamba N., Ruangsittichai J., Chaisiri K., Charoennitiwat V. (2024). Molecular insights versus morphological traits: rethinking identification of the closely related *Angiostrongylus cantonensis* and *Angiostrongylus malaysiensis*. Parasit. Vectors.

[bb0145] Kaewpitoon N., Kaewpitoon S., Pengsaa P. (2008). Food-borne parasitic zoonosis: distribution of trichinosis in Thailand. World J. Gastroenterol..

[bb0150] Kelley J., Elliott T., Beddoe T., Anderson G., Skuce P., Spithill T. (2016). Current threat of triclabendazole resistance in *Fasciola hepatica*. Trends Parasitol..

[bb0155] Khuntikeo N., Titapun A., Loilome W., Yongvanit P., Thinkhamrop B., Chamadol N. (2018). Current perspectives on opisthorchiasis control and cholangiocarcinoma detection in Southeast Asia. Front. Med. (Lausanne).

[bb0160] Kotze A.C., Prichard R.K. (2016). Anthelmintic resistance in *Haemonchus contortus*: history, mechanisms and diagnosis. Adv. Parasitol..

[bb0165] Kusolsuk T., Kamonrattanakun S., Wesanonthawech A., Dekumyoy P., Thaenkham U., Yoonuan T. (2010). The second outbreak of trichinellosis caused by *Trichinella papuae* in Thailand. Trans. R. Soc. Trop. Med. Hyg..

[bb0170] Kusolsuk T., Chaisiri K., Poodeepiyasawad A., Sa-Nguankiat S., Homsuwan N., Yanagida T. (2021). Risk factors and prevalence of taeniasis among the Karen people of Tha song Yang District, Tak Province, Thailand. Parasite.

[bb0175] Le T.H., De N.V., Agatsuma T., Nguyen T.G.T., Nguyen Q.D., McManus D.P. (2008). Human fascioliasis and the presence of hybrid/introgressed forms of *Fasciola hepatica* and *Fasciola gigantica* in Vietnam. Int. J. Parasitol..

[bb0180] Martviset P., Phadungsil W., Na-Bangchang K., Sungkhabut W., Panupornpong T., Prathaphan P. (2023). Current prevalence and geographic distribution of helminth infections in the parasitic endemic areas of rural northeastern Thailand. BMC Public Health.

[bb0185] Martviset P., Phadungsil W., Na-Bangchang K., Sungkhabut W., Panupornpong T., Prathaphan P. (2023). Current prevalence and geographic distribution of helminth infections in the parasitic endemic areas of rural northeastern Thailand. BMC Public Health.

[bb0190] Maurice A.P., Jenkin A., Norton R.E., Hamilton A., Ho Y.H., Tsoulfas G., Hoballah J., Velmahos G., Ho Y.H. (2020). The Surgical Management of Parasitic Diseases.

[bb0195] Morales M.L., Tanabe M.B., White A.C., Lopez M., Bascope R., Cabada M.M. (2021). Triclabendazole treatment failure for *Fasciola hepatica* infection among preschool and school-age children, Cusco. Peru. Emerg. Infect Dis..

[bb0200] Murrell K.D. (2013). Zoonotic foodborne parasites and their surveillance. Rev. Sci. Tech..

[bb0205] Nguyen T., Dermauw V., Dahma H., Bui D.T., Le T.T.H., Phi N.T.T. (2020). Prevalence and risk factors associated with *Clonorchis sinensis* infection in rural communities in northern Vietnam. PLoS Negl. Trop. Dis..

[bb0210] O’Connell E.M., Nutman T.B. (2016). Molecular diagnostics for soil-transmitted helminths. Am. J. Trop. Med. Hyg..

[bb0215] Okello A., Ash A., Keokhamphet C., Hobbs E., Khamlome B., Dorny P. (2014). Investigating a hyper-endemic focus of *Taenia solium* in northern Lao PDR. Parasit. Vectors.

[bb0220] Ortiz J.B., Uys M., Seguino A., Thomas L. (2024). Foodborne helminthiasis. Curr. Clin. Micro. Rpt..

[bb0225] Parija S.C., Poddar A. (2025). Parasitic diagnosis: a journey from basic microscopy to cutting-edge technology. Trop. Parasitol..

[bb0230] Phimpraphai W., Tangkawattana S., Kasemsuwan S., Sripa B. (2018). Social influence in liver fluke transmission: application of social network analysis of food sharing in Thai Isaan culture. Adv. Parasitol..

[bb0235] Prakobwong S., Charoensuk L., Chaipibool S., Chedtabud K., Laothong U., Suwannatrai A. (2025). One health integrated strategies for sustainable control of *Opisthorchis viverrini* infections in rural endemic areas of Thailand. Infect. Dis. Poverty.

[bb0240] Quinn J., Martins N., Cunha M., Higuchi M., Murphy D., Bencko V. (2014). Fragile states, infectious disease and health security: the case for Timor-Leste. J. Hum. Secur..

[bb0245] Saenna P., Hurst C., Echaubard P., Wilcox B., Sripa B. (2017). Fish sharing as a risk factor for *Opisthorchis viverrini* infection: evidence from two villages in North-Eastern Thailand. Infect. Dis. Poverty.

[bb0250] Sahu P.S., Lim Y.A.L., Ngui R., Mahmud R. (2016). Serological evidence of exposure and possible *Taenia solium* larval infection in orang Asli communities of peninsular Malaysia. Trop. Biomed..

[bb0255] Sato M.O., Sato M., Yanagida T., Waikagul J., Pongvongsa T., Sako Y. (2018). Taenia solium, Taenia saginata, Taenia asiatica, their hybrids and other helminthic infections occurring in a neglected tropical diseases’ highly endemic area in Lao PDR. PLoS Negl. Trop. Dis..

[bb0260] Sayasone S., Keiser J., Meister I., Vonghachack Y., Xayavong S., Senngnam K. (2018). Efficacy and safety of tribendimidine versus praziquantel against *Opisthorchis viverrini* in Laos: an open-label, randomised, non-inferiority, phase 2 trial. Lancet Infect. Dis..

[bb0265] Shchelkanov M., Tabakaeva T., Shumenko P., Tabakaev A., Galkina I. (2025). Advantages and limitations of diagnostic procedures for zoonotic helminth infections in feces. J. Helminthol..

[bb0270] Sithithaworn P., Andrews R., De N., Wongsaroj T., Sinuon M., Odermatt P. (2012). The current status of opisthorchiasis and clonorchiasis in the Mekong Basin. Parasitol. Int..

[bb0275] Sota P., Andityas M., Kotepui M., Sripa B. (2024). Prevalence estimates of *Opisthorchis viverrini* and *Clonorchis sinensis* infection in the greater Mekong subregion: a systematic review and meta-analysis. Infect. Dis. Poverty.

[bb0280] Sripa B., Kaewkes S., Intapan P.M., Maleewong W., Brindley P.J. (2010). Food-borne trematodiases in Southeast Asia: epidemiology, pathology, clinical manifestation and control. Adv. Parasitol..

[bb0285] Sripa B., Tangkawattana S., Laha T., Kaewkes S., Mallory F.F., Smith J.F. (2015). Toward integrated opisthorchiasis control in Northeast Thailand: the Lawa project. Acta Trop..

[bb0290] Sripa B., Suwannatrai A.T., Sayasone S., Do D.T., Khieu V., Yang Y. (2021). Current status of human liver fluke infections in the greater Mekong subregion. Acta Trop..

[bb0295] Susilawathi N.M., Suryapraba A., Soejitno A., Asih M., Swastika K., Wandra T. (2020). Neurocysticercosis cases identified at Sanglah hospital, Bali, Indonesia from 2014 to 2018. Acta Trop..

[bb0300] Swe M.M.M., Phyo A.P., Cooper B.S., White N.J., Smithuis F., Ashley E.A. (2023). A systematic review of neglected tropical diseases (NTDs) in Myanmar. PLoS Negl. Trop. Dis..

[bb0305] Tan S., Machrumnizar M. (2023). Fish and food-fatale: food-borne trematode *Opisthorchis viverrini* and cholangiocarcinoma. Helminthologia.

[bb0315] Tidman R., Kanankege K., Bangert M., Abela-Ridder B. (2023). Global prevalence of 4 neglected foodborne trematodes targeted for control by WHO: a scoping review to highlight the gaps. PLoS Negl. Trop. Dis..

[bb0320] Toribio L.M., Bustos J.A., Garcia H.H. (2024). From laboratory to clinical practice: an update of the immunological and molecular tools for neurocysticercosis diagnosis. Front. Parasitol..

[bb0325] Utzinger J., Brattig N., Leonardo L., Zhou X., Bergquist R. (2015). Progress in research, control and elimination of helminth infections in Asia. Acta Trop..

[bb0330] Velev V., Vutova K., Pavlova M., Mangarov I. (2020). A case of fasciolosis in a 11-year-old Syrian immigrant boy. J. Trop. Pediatr..

[bb0335] Wattanawong O., Iamsirithaworn S., Kophachon T., Nak-Ai W., Wisetmora A., Wongsaroj T. (2021). Current status of helminthiases in Thailand: a cross-sectional, nationwide survey, 2019. Acta Trop..

[bb0340] World Health Organization (2012).

